# The diagnostic value of a nomogram based on enhanced CT radiomics for differentiating between intrahepatic cholangiocarcinoma and early hepatic abscess

**DOI:** 10.3389/fmolb.2024.1409060

**Published:** 2024-08-23

**Authors:** Meng-chen Yang, Hai-yang Liu, Yan-ming Zhang, Yi Guo, Shang-yu Yang, Hua-wei Zhang, Bao Cui, Tian-min Zhou, Hao-xiang Guo, Dan-wei Hou

**Affiliations:** Department of Medical Imaging, Shangluo Central Hospital, Shangluo, China

**Keywords:** early hepatic abscess, intrahepatic cholangiocarcinoma, radiomics, enhancement scanning, nomogram

## Abstract

**Objective:**

This study aimed to investigate the value of a CT-enhanced scanning radiomics nomogram in distinguishing between early hepatic abscess (EHA) and intrahepatic cholangiocarcinoma (ICC) and to validate its diagnostic efficacy.

**Materials and Methods:**

Clinical and imaging data on 112 patients diagnosed with EHA and ICC who underwent double-phase CT-enhanced scanning at our hospital were collected. The contours of the lesions were delineated layer by layer across the three phases of CT scanning and enhancement using 3D Slicer software to define the region of interest (ROI). Subsequently, the contours were merged into 3D models, and radiomics features were extracted using the Radiomics plug-in. The data were randomly divided into training (n = 78) and validation (n = 34) cohorts at a 7:3 ratio, using the R programming language. Standardization was performed using the Z-score method, and LASSO regression was used to select the best λ-value for screening variables, which were then used to establish prediction models. The rad-score was calculated using the best radiomics model, and a joint model was constructed based on the rad-score and clinical scores. A nomogram was developed based on the joint model. The diagnostic efficacy of the models for distinguishing ICC and EHA was assessed using receiver operating characteristic (ROC) curve and area under the curve (AUC) analyses. Calibration curves were used to evaluate the reliability and accuracy of the nomograms, while decision curves and clinical impact curves were utilized to assess their clinical value.

**Results:**

Compared with the ICC group, significant differences were observed in clinical data and imaging characteristics in the EHA group, including age, centripetal enhancement, hepatic pericardial depression sign, arterial perfusion abnormality, arterial CT value, and arteriovenous enhancement (*p* < 0.05). Logistic regression analysis identified centripetal enhancement, hepatic pericardial depression sign, arterial perfusion abnormality, arterial CT value, and arteriovenous enhancement as independent influencing factors. Three, five, and four radiomics features were retained in the scanning, arterial, and venous phases, respectively. Single-phase models were constructed, with the radiomics model from the arterial phase demonstrating the best diagnostic efficacy. The rad-score was calculated using the arterial-phase radiomics model, and nomograms were drawn in conjunction with the clinical model. The nomogram based on the combined model exhibited the highest differential diagnostic efficacy between EHA and ICC (training cohort: AUC of 0.972; validation cohort: AUC of 0.868). The calibration curves indicated good agreement between the predicted and pathological results, while decision curves and clinical impact curves demonstrated higher clinical utility of the nomograms.

**Conclusion:**

The CT-enhanced scanning radiomics nomogram demonstrates high clinical value in distinguishing between EHA and ICC, thereby enhancing the accuracy of preoperative diagnosis.

## Introduction

Hepatic abscesses are relatively common in clinical practice, and their imaging characteristics vary depending on the stage of the disease. In recent years, the imaging features of early hepatic abscess (EHA) have become increasingly atypical, possibly due to antibiotic misuse (He et al., 2022). The absence of central uniform necrotic areas, ring target signs, or gas–liquid interfaces within the lesions makes it challenging to definitively diagnose EHA, often leading to misdiagnosis as tumorous lesions (Zhuo et al., 2021; Khim et al., 2019). Intrahepatic cholangiocarcinoma (ICC), the second most common primary malignant liver tumor, arises from the epithelial cells of the bile ducts. It exhibits an insidious onset, accounting for approximately 15% of liver cancer cases. ICC presents with various imaging features, including clinical fever and honeycomb changes in some cases (Hu et al., 2022; Entezari and Riaz, 2020). Therefore, an accurate preoperative diagnosis of ICC significantly influences treatment options and patient prognosis. Radiomics, a machine learning-based method introduced by Kumar in 2012, allows for the extraction of high-throughput radiomics features (>100 features) from conventional images to quantify lesions. This emerging technology has gained widespread use in recent years (Kumar et al., 2012; Chassagnon et al., 2023). However, there are limited reports in the literature regarding the application of CT-enhanced scanning radiomics nomograms for differentiating and identifying liver lesions. This study aims to establish CT-enhanced scanning radiomics nomograms and assess their clinical utility in distinguishing EHA from ICC.

## Materials and methods

### General information

We conducted a retrospective analysis of 53 patients diagnosed with EHA and 59 patients diagnosed with ICC, who underwent dual-phase CT-enhanced scanning of the abdomen at our department between January 2019 and December 2023. The diagnoses were confirmed by pathology, and clinical data and CT imaging features were collected for each patient. In the EHA group, there were 30 male and 23 female individuals with a mean age of 60.4 ± 12.9 years. In the ICC group, there were 33 male and 26 female individuals with a mean age of 65.3 ± 12.2 years. The inclusion criteria were as follows: (Ⅰ) all patients were first-time admissions and underwent CT-enhanced scanning examination; (Ⅱ) patients with ICC and EHA who had not received any treatment; and (Ⅲ) no history of liver surgery. The exclusion criteria were as follows: (Ⅰ) patients who had undergone drainage of hepatic abscesses; (Ⅱ) patients who had undergone radiotherapy for other types of cancer; and (Ⅲ) patients without pathological results. The collected clinical data and imaging characteristics comprised the basic dataset, which was then randomly divided into a training cohort (n = 78) and a validation cohort (n = 34) at a ratio of 7:3 using the R programming language.

### CT examination methods

Abdominal plain scanning and dual-phase enhancement scanning examinations were conducted using Philips IQon Spectral CT/Brilliance 64-row spiral CT and GE 256-row Revolution CT equipment. The scanning parameters included a matrix size of 512*512, axial thin-layer thickness of 0.9 mm, and a scanning range extending from the level of the diaphragm to the level of the anterior superior spine of the iliac spine. The contrast agent iophorol was administered at a dosage of 2 mg/kg with an injection flow rate of 3.5 mL/s through the median vein of the elbow. Scans were performed in the arterial phase at 25 s and in the venous phase at 38 s after the injection of the contrast material.

### Measurement standards and image analysis

CT values were measured during the scanning, arterial, and venous phases for all 112 patients. To ensure consistency in the measurement location and level, all images were synchronized across multiple phases using picture archiving and communication systems (PACS). The measurement area was ensured to be situated within the substance of the lesion, and the maximum diameter of the lesion was measured at the level with the best axial image morphology. A double-blinded review of the CT images of all patients was performed independently by two attending physicians from the Department of Imaging. In cases of disagreement, a third experienced chief physician adjudicated the decision. The following aspects were discussed: lesion site (right lobe of the liver and left lobe of the liver), lesion morphology (regular and irregular), centripetal enhancement (have and no), hepatic pericardial depression sign (have and no), arterial-phase perfusion abnormality (have and no), foveal changes (have and no), maximum diameter of the lesion, CT value of the plain and arterial and venous phases, magnitude of enhancement in the arterial phase, and difference in the enhancement of arterial and venous phases (12 indexes), and the maximum diameter of the lesion and the plain phase. The maximum diameter of the lesion in the plain phase and the CT value in each phase (plain, arterial, and venous) were averaged by the two attending physicians.

### Radiomics data acquisition

The sweeping arterial and venous images of 112 patients were exported in DICOM format and imported into 3D Slicer software. Regions of interest (ROIs) were delineated layer by layer along the edges of the lesions by the two attending physicians mentioned above. These ROIs were then fused into a 3D volume of interest (VOI) using the “Fill between slices” function within the software program. Subsequently, the Radiomics plug-in was utilized to resample the images for normalization (voxel: 1 mm × 1 mm × 1 mm) and discretization of gray values (bin width: 25) to mitigate the effects of heterogeneity (Leijenaar et al., 2015). This process aimed to reduce variability in radiomics features due to differences in image resolution and intensity. Following normalization and discretization, the original features and higher-order features based on wavelet transform were extracted. A total of 851 radiomics features were extracted from each of the three phases. These features included 14 morphological features, 18 first-order features, 65 texture features, and 754 higher-order features based on the wavelet transform.

### Dimensionality reduction screening of radiomics features and calculation of the rad-score

First, the ROIs outlined by the two attending physicians were analyzed for consistency, and the interclass correlation coefficient was calculated. Data on variables with an interclass correlation coefficient > 0.75 were retained. Next, the Z-score method was utilized to standardize all the data, ensuring uniformity in scale across variables. Subsequently, the standardized data were subjected to feature selection using LASSO regression with a 10-fold cross-validation to determine the optimal penalty coefficient λ that resulted in the smallest error. Redundant features with correlation coefficients |r| > 0.8 were excluded using Spearman’s correlation analysis to minimize multicollinearity. The final retained feature coefficients and their correlation data were utilized to calculate the rad-score for each phase and construct a logistic regression model. The rad-score was calculated as follows: Rad-score = I + 1β*1R + 2β*2R + ... (I represents the cutoff value, β represents the value of each characteristic coefficient, and R represents the value of each characteristic).

### Statistical methods

R 4.3.1 software was utilized to assess the normality of measurements in the dataset using the Kolmogorov–Smirnov test. Levene’s test was used to assess the homogeneity of variances. For normally distributed data, *t*-tests were conducted (homogeneous variance) or Welch’s *t*-tests were applied (heterogeneity of variance). Non-normally distributed data were analyzed using Wilcoxon’s rank-sum test, with results expressed as x ± s. Categorical data were analyzed using the chi-squared test or Fisher’s exact test for special variables, with frequencies reported as n (%). Single-factor and multi-factor logistic regression analyses were performed to identify independent influencing factors and construct various models in both the training and validation cohorts. Receiver operating characteristic (ROC) curves were utilized to analyze and calculate the cutoff value, Jordon’s index, sensitivity, specificity, and area under the curve (AUC) with a 95% confidence interval (CI) for each model. Based on the joint model, column line graphs were generated using R software. Calibration curves were plotted after 1,000 repetitions using the bootstrap self-sampling method to visually evaluate the predictive performance of the column line graphs. Decision curves and clinical impact curves were plotted to evaluate the clinical utility of the column line graphs. Statistical significance was set at *p* < 0.05.

## Results

### Comparison of general information and CT features

**FIGURE 1:**
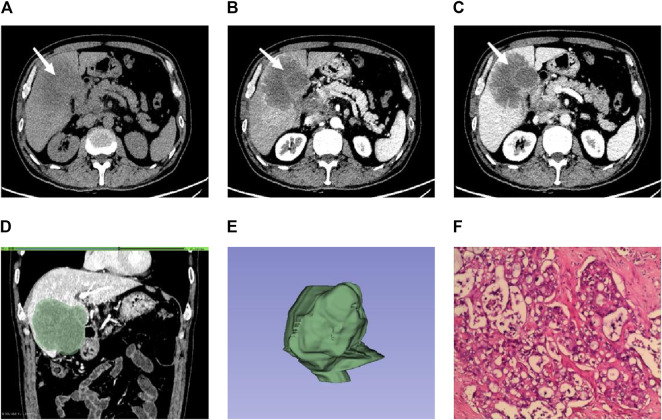
Male individual, 65 years old, ICC **(A–C)**. The lesions were located in the right lobe of the liver (white arrow). The enhancement was uneven in the arterial phase. The enhancement in the venous phase was high and showed centripetal enhancement **(D)**. ROI is outlined on the largest level of the lesion in the venous phase **(E)**. 3D view of the lesion **(F)**. Pathological findings showed irregular glandular tubular and strip-like interstitial infiltration with obvious cell atypia, which was consistent with moderately differentiated intrahepatic cholangiocarcinoma (HE × 100).

**FIGURE 2:**
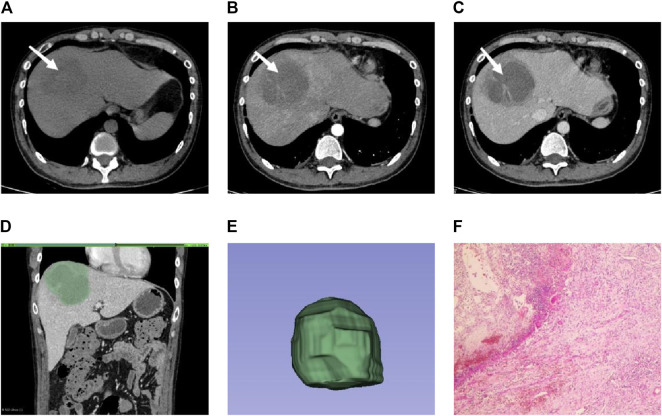
Male individual, 36 years old, EHA **(A–C)**. The lesions were located in the right lobe of the liver (white arrow). Abnormal perfusion could be observed in the arterial stage. The degree of enhancement in the venous stage was not high **(D)**. ROI is outlined on the largest level of the lesion in the venous phase **(E).** 3D view of the lesion **(F)**. The pathology showed extensive neutrophil infiltration with necrosis, consistent with hepatic abscess (HE × 100).

Of the 14 indicators in the basic dataset, gender, lesion location, lesion morphology, honeycomb changes, maximum lesion diameter, CT value in the plain phase, CT value in the venous phase, and amplitude of enhancement in the arterial phase were not statistically significant (*p* > 0.05) in ICC (Figures 1A–F) and EHA (Figures 2A–F), whereas the clinical information and imaging features of age, centripetal enhancement, hepatic pericardial depression sign, arterial-phase perfusion abnormality, arterial-phase CT value, and arterial vein-phase enhancement difference were statistically significant (*p* < 0.05) in all six indicators. Specifically, patients with hepatic abscess were generally younger, ICC had more centripetal enhancement and hepatic pericardial depression sign, arterial-phase perfusion abnormality was more common in hepatic abscess, arterial-phase enhancement of ICC patients was slightly higher than that of patients with EHA, and arteriovenous enhancement difference of EHA was higher than that of ICC (Table 1).

**TABLE 1:** Characteristics of patients in the training and validation cohorts.

Characteristic	Training cohort				Validation cohort			
	ICC (n = 42)	EHA (n = 36)	t/Z/χ2	*p*-value	ICC (n = 17)	EHA (n = 17)	t/Z/χ^2^	*p-*value
Gender [n(%)]			0.320	0.572			0.472	0.492
Male	23 (55)	22 (61)			10 (59)	8 (47)		
Female	19 (45)	14 (39)			7 (41)	9 (53)		
Age	65.2 ± 11.8	58.1 ± 14.1	2.397	0.018	65.4 ± 12.5	62.7 ± 11.8	0.650	0.520
Diseased region [n(%)]			3.243	0.072				0.118*
Left lobe	16 (38)	7 (20)			7 (41)	2 (12)		
Right lobe	26 (62)	29 (80)			10 (59)	15 (88)		
Morphology [n(%)]			2.226	0.136			4.371	0.037
Regular	14 (33)	18 (50)			4 (24)	10 (58)		
Irregular	28 (67)	18 (50)			13 (76)	7 (42)		
CR [n(%)]			34.552	<0.001			9.663	0.002
Have	35 (83)	6 (17)			12 (71)	3 (18)		
No	7 (17)	30 (83)			5 (29)	14 (82)		
LED [n(%)]			28.627	<0.001			15.07	0.001
Have	27 (64)	2 (6)			12 (71)	1 (6)		
No	15 (36)	34 (94)			5 (29)	16 (94)		
AAPP [n(%)]			6.980	0.008			4.636	0.031
Have	11 (26)	20 (56)			3 (18)	9 (53)		
No	31 (74)	16 (44)			14 (82)	8 (47)		
Honeycomb change [n(%)]			0.896	0.344			0.486	0.486
Have	11 (26)	13 (36)			6 (35)	8 (47)		
No	31 (74)	23 (64)			11 (65)	9 (53)		
MLD ( $\bar{x}$ ±s, cm)	6.89 ± 3.85	6.14 ± 2.88	0.981	0.340	6.91 ± 3.61	5.02 ± 2.70	183.0	0.193
PSPCTV ( $\bar{x}$ ±s, HU)	33.6 ± 6.5	31.3 ± 7.9	1.393	0.162	33.8 ± 6.13	33.6 ± 10.6	0.061	0.953
APCTV ( $\bar{x}$ ±s, HU)	53.4 ± 9.2	48.4 ± 11.4	2.138	0.033	52.3 ± 12.6	51.9 ± 13.6	0.082	0.935
VPCTV ( $\bar{x}$ ±s, HU)	65.2 ± 14.5	64.0 ± 18.1	0.306	0.757	60.3 ± 12.6	70.5 ± 21.4	−1.699	0.101
APRA ( $\bar{x}$ ±s, HU)	19.9 ± 9.0	17.1 ± 9.2	890.0	0.179	18.5 ± 12.2	18.4 ± 12.1	0.046	0.964
APED ( $\bar{x}$ ±s, HU)	−11.7 ± 10.2	−15.7 ± 12.7	969.5	0.032	−7.9 ± 5.5	−18.6 ± 15.5	215.0	0.015

ICC, intrahepatic cholangiocarcinoma; EHA, early hepatic abscess; CR, centripetal reinforcement; LED, liver envelope depression; AAPP, abnormal arterial phase perfusion; MLD, maximum lesion diameter; PSPCTV, plain scan phase CT value; APCTV, arterial phase CT value; VPCTV, venous phase CT value; APRA, arterial phase reinforcement amplitude; APED, arteriovenous phase enhancement difference.

Note: * is Fisher’s exact test.

### Modeling of clinical and imaging data

The above statistically significant variables (age, centripetal enhancement, hepatic pericardial depression sign, arterial perfusion abnormality, arterial CT value, and arteriovenous enhancement difference) were included in the binary logistic regression analysis. The results showed that centripetal enhancement, hepatic pericardial depression sign, arterial-phase CT value, and arteriovenous-phase enhancement difference were independent influences; age and abnormal arterial-phase perfusion were not independent factors (*p* > 0.05); and centripetal enhancement was the most sensitive factor (*p* < 0.001) (Table 2).

**TABLE 2:** Multivariate logistic regression analysis of the training cohort.

Characteristic	OR (95% CI)	*β*	Wald	*p*-value
Age	0.98 (0.92–1.04)	−0.022	0.557	0.500
CR	13.71 (3.39–68.6)	2.618	12.138	<0.001
LED	10.76 (2.02–87.1)	2.370	6.620	0.010
AAPP	0.52 (0.09–2.61)	−0.697	0.707	0.400
APCTV	0.94 (0.87–1.01)	−0.061	2.769	0.042
APED	0.97 (0.91–1.04)	−0.025	3.165	0.039
Intercept		−2.927	0.629	0.476

CR, centripetal reinforcement; LED, liver envelope depression; AAPP, abnormal arterial phase perfusion; APCTV, arterial phase CT value; APED, arteriovenous phase enhancement difference.

### Radiomics feature extraction and nomogram plot

**FIGURE 3:**
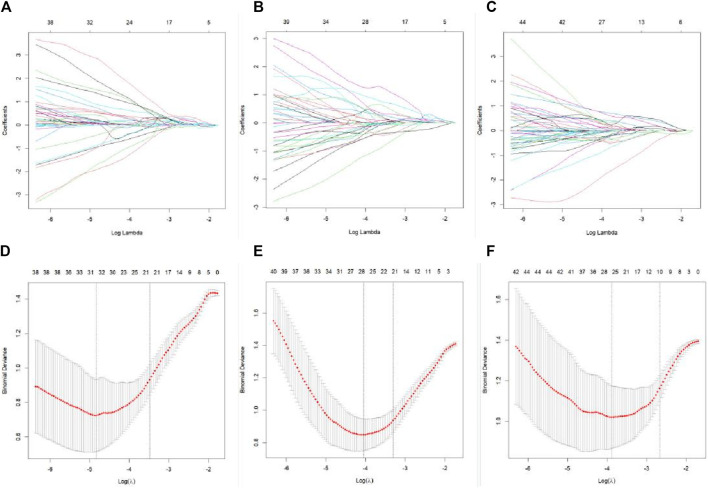
LASSO regression feature screening graph. A 10-fold cross-verified LASSO regression was used to screen the radiomics features, and the optimal parameter λ was selected **(A–C)**. Curves of the changes in the radiomics feature coefficients with λ in the plain scan phase, arterial stage, and venous stage models, respectively. The dotted line indicates the location of the selected optimal λ **(D–F)**. Curves of the mean square error of the model in plain scan phase, arterial phase, and venous phase with λ, respectively. The dashed line represents standard deviation of 1 and the location of the selected optimal λ.

**FIGURE 4:**
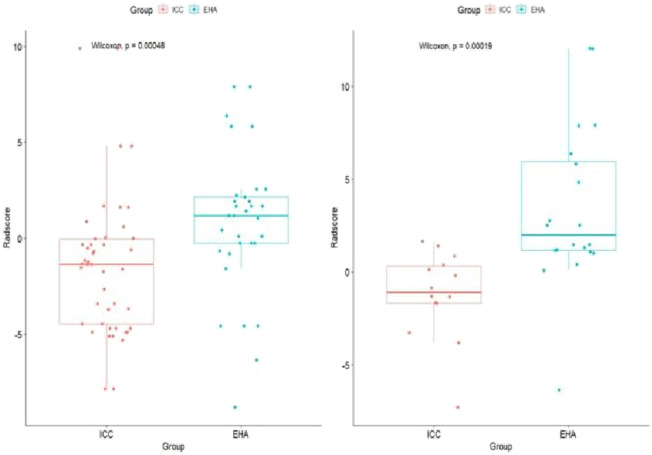
Rad-score boxplot to distinguish ICC and EHA. The rad-score of the ICC group was significantly lower than that of the EHA group in both the training and validation cohorts.

**FIGURE 5:**
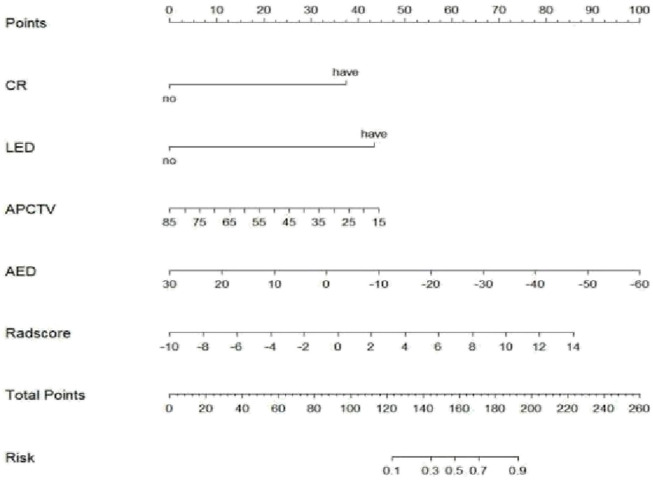
Nomogram of the arterial phase rad-score combined with the clinical model in differential diagnosis of ICC and EHA. CR, centripetal reinforcement; LED, liver envelope depression; APCTV, arterial-phase CT values; AED, arteriovenous enhancement difference.

The 3D Slicer Radiomics plug-in was utilized to extract 861 radiomics features from each of the three-phase images. These included 14 morphological features, 18 first-order features, 75 texture features, and 754 higher-order features based on the wavelet transform. The base pixel comprised 14 gray-level dependence matrix (GLDM), 5 neighborhood gray-tone difference matrix (NGTDM), 16 gray-level size zone matrix (GLSZM), 38 gray-level co-occurrence matrix (GLCM), and 32 gray-level run length matrix (GLRLM). Radiomics data typically have high-dimensional features, and LASSO regression, by applying L1 regularization, can handle these high-dimensional data, reducing model complexity and preventing overfitting (Lambin et al., 2012). LASSO regression and 10-fold cross-validation were used to select the best penalty factor λ with the lowest error for both the imaging data and clinical data (Figure 3). Ultimately, a total of 12 features were retained in each phase (Table 3). The arterial-phase radiomics model demonstrated optimal efficacy among the radiomics models in each phase (AUC = 0.905; 95% CI: 0.856–0.943). Consequently, arterial-phase radiomics data were selected for the calculation of the rad-score. The rad-score equation derived from the arterial-phase radiomics data was as follows: Rad-score = 0.091 + 3.057wavelet-LLL_glcm_MCC+1.508wavelet-LLH_firstorder_Skewness +1.652 wavelet-LLH_glrlm_ShortRunHighGrayLevelEmphasis + 0.624 original_shape_Flatness-0.589 * wavelet-HLL_glszm_Small AreaEmphasis. The analysis of sample data (Figure 4) revealed that the rad-score of the ICC group was significantly lower than that of the EHA group in both the training (Z = 397, *p* < 0.001) and the validation cohorts (Z = 33, *p* < 0.001). Subsequently, the rad-score was merged with the clinical model to construct the nomogram model and plot the nomogram (Figure 5).

**TABLE 3:** Final screening characteristics and coefficients.

Intercept	Phases and characteristics	Name of the radiomics feature	Coefficient
−0.085	Plain scan phase	wavelet-HLH_firstorder_Skewness	−0.101
		wavelet-LLH_firstorder_Maximum	0.187
		wavelet-HHL_glcm_MCC	0.329
0.091	Arterial phase	wavelet-LLL_glcm_MCC	3.057
		wavelet-LLH_firstorder_Skewness	1.508
		wavelet-LLH_glrlm_ShortRunHighGrayLevelEmphasis	1.652
		original_shape_Flatness	0.624
		wavelet-HLL_glszm_SmallAreaEmphasis	−0.589
−0.142	Venous phase	wavelet-LLL_glcm_Idmn	−0.452
		wavelet-LHL_glrlm_RunVariance	−0.309
		wavelet-HLH_firstorder_Mean	−0.069
		wavelet-HHH_glcm_Imc1	0.299

Note: + and - in the coefficient represent addition and subtraction in the formula, respectively.

### Evaluation of the diagnostic efficacy of each model

**FIGURE 6:**
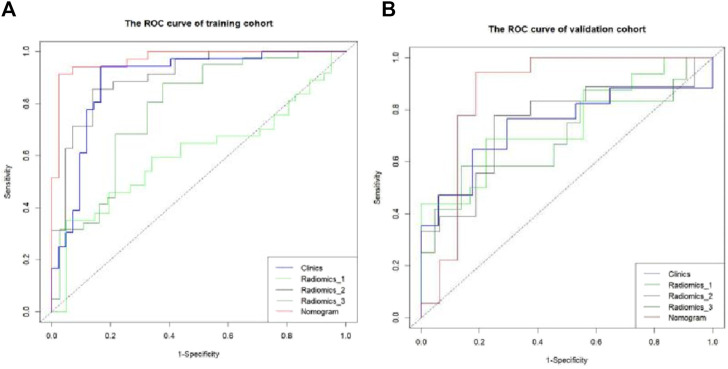
ROC curve of differential diagnosis of each model in the training and validation cohorts. Radiomics_1, Radiomics_2, and Radiomics_3 represent the rad-score model in the plain scan phase, arterial phase, and venous phase, respectively. Nomogram stands for the combined model.

**FIGURE 7:**
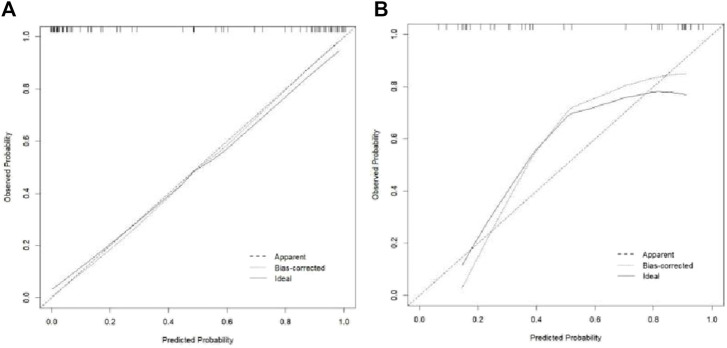
Calibration curve of the training and validation cohorts.

**FIGURE 8:**
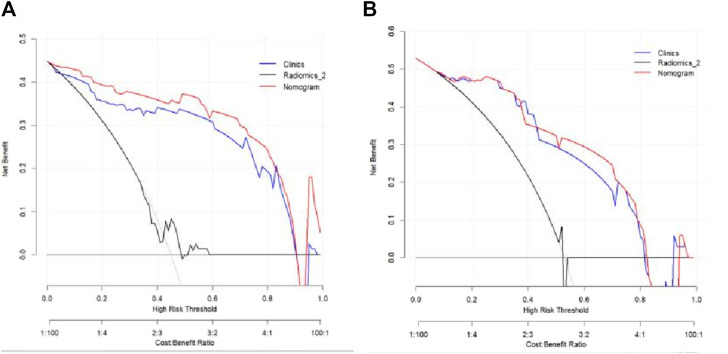
Decision curves of the clinical model, Radiomics_2 (arterial phase rad-score model), and nomogram model in the training and validation cohorts.

**FIGURE 9:**
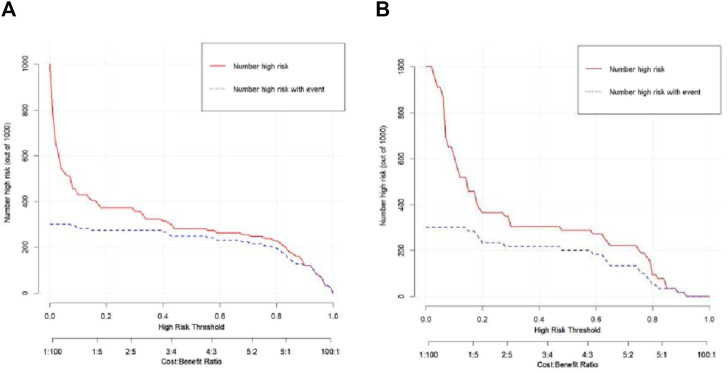
Clinical impact curve of the nomogram in the training and validation cohorts.

The Hosmer–Lemeshow test results for each model in both the training and validation cohorts indicated *p* > 0.05, suggesting that the models were well fitted. ROC curves were plotted based on the predictive probabilities of each model in both cohorts (Figure 6). The corresponding AUC values (95% CI), specificity, sensitivity, cutoff value, and Jordon’s index of each model were obtained and are given in Table 4. In the training cohort, the clinical model, arteriomics model, and nomogram model demonstrated AUC values (95% CI) of 0.890 (0.847–0.932), 0.905 (0.856–0.943), and 0.972 (0.958–1), respectively. Specificity and sensitivity for each model were 0.833 and 0.944; 0.861 and 0.857; and 0.977 and 0.914, respectively. In the validation cohort, the clinical model, arteriomics model, and nomogram model exhibited AUC values (95% CI) of 0.740 (0.682–0.763), 0.753 (0.706–0.792), and 0.868 (0.814–0.901), respectively. Specificity and sensitivity for each model were 0.823 and 0.647; 0.750 and 0.778; and 0.813 and 0.944, respectively. Calibration curves of the column–line graphs for both the training and validation cohorts were plotted to assess the agreement between the predicted probabilities of the column–line graphs and the pathological results (Figure 7). The high agreement indicated good predictive performance. Decision curves demonstrated that the column–line diagram had a wider range of risk thresholds than other models, resulting in a higher net clinical benefit (Figure 8). Furthermore, the clinical impact curve indicated that the column–line diagram had superior clinical application value (Figure 9).

**TABLE 4:** Diagnostic efficiency of each model in the training and validation cohorts.

Characteristic	Intercept	Jordon index	Sensitivity	Specificity	AUC (95% CI)
Training cohort (n = 78)					
Clinics	0.268	0.778	0.944	0.833	0.890 (0.847–0.932)
Radiomics_1	0.638	0.303	0.351	0.951	0.606 (0.549–0.651)
Radiomics_2	0.508	0.718	0.857	0.861	0.905 (0.856–0.943)
Radiomics_3	0.416	0.499	0.878	0.622	0.780 (0.727–0.826)
Nomogram	0.673	0.891	0.914	0.977	0.972 (0.958–1)
Validation cohort (n = 34)					
Clinics	0.473	0.470	0.647	0.823	0.740 (0.682–0.763)
Radiomics_1	0.442	0.465	0.688	0.778	0.747 (0.697–0.787)
Radiomics_2	0.469	0.528	0.778	0.750	0.753 (0.706–0.792)
Radiomics_3	0.464	0.447	0.583	0.863	0.697 (0.651–0.724)
Nomogram	0.417	0.757	0.944	0.813	0.868 (0.814–0.901)

Note: Radiomics_1, Radiomics_2, and Radiomics_3 represent the rad-score model in the plain scan phase, arterial phase, and venous phase, respectively. The nomogram stands for the combined model.

## Discussion

At present, there are many tumor-related diagnostic methods, such as nanotechnology in tumor liquid biopsy and nanomaterial-assisted metabolic analysis in clinical application (Zhu et al., 2024; Yang et al., 2022). However, in recent years, the incidence of hepatic abscesses has increased, attributed to factors such as the aging population, increased prevalence of diabetes mellitus, and bile duct diseases (Wang et al., 2023). EHAs often present atypically in terms of imaging performance during the abscess formation stage, leading to difficulty in distinguishing them from liver tumors (Priyadarshi et al., 2022). Similarly, ICC exhibits mixed imaging manifestations, contributing to a higher misdiagnosis rate in clinical settings (Ke et al., 2023). Both EHA and ICC can display irregular lesion morphology, centripetal enhancement, arterial-phase perfusion abnormalities, and honeycomb changes. However, the clinical treatments for these conditions are vastly different. Misdiagnosing a hepatic abscess as ICC and performing clinical drainage can increase the risk of cancer cell dissemination, significantly impacting patient prognosis. In our study, we collected clinical and imaging data from 112 cases and observed several noteworthy findings. Patients with EHA were generally younger than those with ICC, consistent with existing literature reports (Devulapalli et al., 2018). Additionally, ICC exhibited centripetal enhancement compared to EHA, possibly due to iodine contrast agent leakage into the vascular space and slower penetration of the fibrous tissue component of ICC (Iavarone et al., 2013; Wang et al., 2020; Tarnoki et al., 2021). The presence of hepatic pericardial depression sign was a typical malignant indicator observed only in ICC, while perfusion abnormalities in the arterial phase were predominantly observed in hepatic abscesses, aligning with previous reports (Li et al., 2023). Furthermore, the magnitude of enhancement in the arterial phase was slightly higher in ICC than in EHA, and the difference between arterial and venous enhancement was greater in EHA than in ICC, consistent with prior studies (Yu et al., 2014; Oh et al., 2019). However, gender, lesion location, lesion morphology, foveal changes, maximum lesion diameter, CT values in the plain and venous phases, and enhancement amplitude in the arterial phase were not statistically significant in our study. Notably, foveal changes are often perplexing in clinical practice and can contribute to misdiagnosis, highlighting the importance of cautious interpretation.

Radiomics has undergone rapid development in the past decade and has found widespread application in the study of various systemic diseases. For instance, it has shown high effectiveness in predicting IDH status in glioma and EGFR mutations in lung cancer (Bogani et al., 2017). Based on CT radiomics parameters, it is also possible to distinguish radiation pneumonitis from immune pneumonitis. These parameters analyze the texture features, shapes, densities, and other information from CT images, helping doctors differentiate between different types of pneumonitis on imaging. Additionally, radiomics parameters can predict PD-L1 and CD8 expression levels, which is crucial for guiding immunotherapy. Through these parameters, doctors can more accurately assess the tumor microenvironment, develop personalized treatment plans, and improve treatment efficacy (Wen et al., 2021; Qiu et al., 2022; Fan et al., 2024). By extracting a large number of quantitative features from images and analyzing the distribution and relationship of pixel intensities, radiomics quantifies the biological characteristics of a disease. This objective assessment of lesion homogeneity is invaluable for accurate diagnosis and prognosis (Yardımcı et al., 2020). Radiomics possesses robust capabilities for objective data mining and quantification (Nie et al., 2019), which are increasingly vital in disease research. The features extractable through radiomics encompass three main categories: first-order features based on grayscale and shape, second-order features based on texture, and higher-order features (such as filtering and wavelet transforms). These categories can yield hundreds or thousands of small features. However, it is not necessarily true that more features lead to better outcomes. Excessive variables may result in overfitting of the model, necessitating feature selection to ensure the model robustness. Hence, the extracted features require careful filtering. Despite the quantitative information that radiomics offers, which is often imperceptible to the naked eye, its clinical application has been limited due to technical challenges. These include issues related to improving reproducibility, data openness and sharing, and low interpretability (Papadimitroulas et al., 2021). Consequently, the clinical translation of radiomics continues to encounter significant hurdles.

In this study, 10-fold cross-validation LASSO regression was used to screen the features, where the coefficients of non-significant variables were compressed to 0 by adjusting the parameter λ. Ultimately, a total of 12 features were selected, with 11 of them being higher-order features based on the wavelet transform. This observation underscores the significant role of wavelet features in identifying the nature of the lesion. As reported in Atto et al. (2013), wavelet features can categorize image attributes into different subsets and use distinct algorithms for each subset to enhance image information and emphasize details, thus offering higher diagnostic value than other features. Wavelet filtering, a method for image noise reduction, diminishes smoothing and decomposes image information in the spatial frequency domain, thereby preserving more detailed image features (Gungor et al., 2021; Zhang et al., 2022). Consequently, it finds wide application in digital image processing. In this study, the nomogram model demonstrated significant value in distinguishing EHA from ICC. By integrating the clinical characteristics and imaging parameters of patients, the nomogram model effectively predicts and differentiates these two diseases, providing accurate diagnostic information. The nomogram model generates individualized risk predictions by combining multiple variables (such as age, gender, tumor size, and radiomics features), enhancing diagnostic accuracy and reliability. Consequently, it enables timely and appropriate therapeutic interventions, which helps reduce misdiagnosis and overtreatment, thereby improving patient prognosis and survival rates. Therefore, as a comprehensive assessment tool, the nomogram model has broad application prospects in liver disease imaging diagnosis and deserves further promotion and application.

This study is subject to the following four limitations: (Ⅰ) the small sample size may introduce bias into the statistical results of the data; (Ⅱ) the wide time span of the data collected poses challenges in ensuring the accuracy of the images measured by the three models; (Ⅲ) in this study, the samples were randomly divided into groups at a 7:3 ratio; however, the optimal grouping method should be based on a 5-fold cross-validation to ensure the reliability and generalizability of the model; and (IV) the study exclusively utilized the logistic model algorithm, neglecting the exploration of other higher-order algorithms such as support vector machines and naive Bayes. To validate the results and overcome these limitations, a multi-center, multi-algorithm, and large-sample study should be conducted.

## Conclusion

In summary, CT-enhanced scanning-based radiomics offers a novel approach to clinical practice, with its nomogram model proving to be more effective in distinguishing between ICC and EHA. This improvement in accuracy aids in enhancing preoperative diagnosis and provides valuable data support for selecting subsequent clinical treatments. Moreover, the visualization properties of the nomogram confer significance for clinical promotion, facilitating its application in clinical settings.

## Data Availability

The raw data supporting the conclusion of this article will be made available by the authors, without undue reservation.
